# Supplementary material for: Competitive hierarchies in bryozoan assemblages mitigate network instability by keeping short and long feedback loops weak
Source: Commun Biol. 2023 Jul 4;6:690. doi: 10.1038/s42003-023-05060-1 (PMC10319822; doi:10.1038/s42003-023-05060-1)
Supplement: Supplementary file 2 — Supplemental Material [file 42003_2023_5060_MOESM2_ESM.pdf]

Supplementary Material for:

Competitive hierarchies in bryozoan assemblages mitigate network instability by keeping short and long feedback loops weak

Franziska Koch[1,2,*†], Anje-Margriet Neutel[3†], David K. A. Barnes[3], Katja Tielbörger[1], Christiane Zarfl[1], Korinna T. Allhoff[1,2,4]

Affiliations:

[1]University of Tübingen, Tübingen, Germany

[2]University of Hohenheim, Stuttgart, Germany

[3]British Antarctic Survey, Cambridge, United Kingdom

[4]KomBioTa – Center for Biodiversity and Integrative Taxonomy, University of Hohenheim & State Museum of Natural History, Stuttgart, Germany

†These authors contributed equally to this work

*Corresponding author. Email: franziska.koch@uni-hohenheim.de, korinna.allhoff@uni-hohenheim.de

Supplementary Note 1:

Sensitivity to the choice of cost values

Our estimation of energy loss rates from observations of competitive outcome is based on the assumption of fixed amounts of biomass loss for each type of competitive outcome. We performed a sensitivity analysis to ensure that our results do not depend on the choice of cost-values. The cost-values chosen in the main part of our analysis were p_W= -0.1, p_L= -0.9, p_D=-0.2. In addition to this, the analysis was repeated in four different scenarios:

1. p_L+: The ratio of win to loss costs is decreased: p_W= -0.1, p_L= -0.4, p_D=-0.2
2. p_L-: The ratio of win to loss costs was increased: p_W= -0.1, p_L= -1.2, p_D=-0.2
3. p_D+: The cost of a draw was increased: p_W= -0.1, p_L= -0.9, p_D=-0.4
4. $p_x \cdot 2$: Same ratios, but all costs are doubled: p_W= -0.2, p_L= -1.8, p_D=-0.4

These scenarios were chosen to cover different extreme cases for possible relationships between parameter values. While varying the cost-values did impact stability, measured as s^*, we found that this did not affect the qualitative results of our analysis. All empirical systems remained unstable, in all scenarios. Also, their relative stability compared to other empirical systems remained mostly unchanged. Those networks that were most unstable in the original analysis were also most unstable in the other scenarios (Supplementary Fig 2). Furthermore, the relationship between s^* and the mean weight of 2-link loops was not affected (Supplementary Fig 3).

Supplementary Figures

a

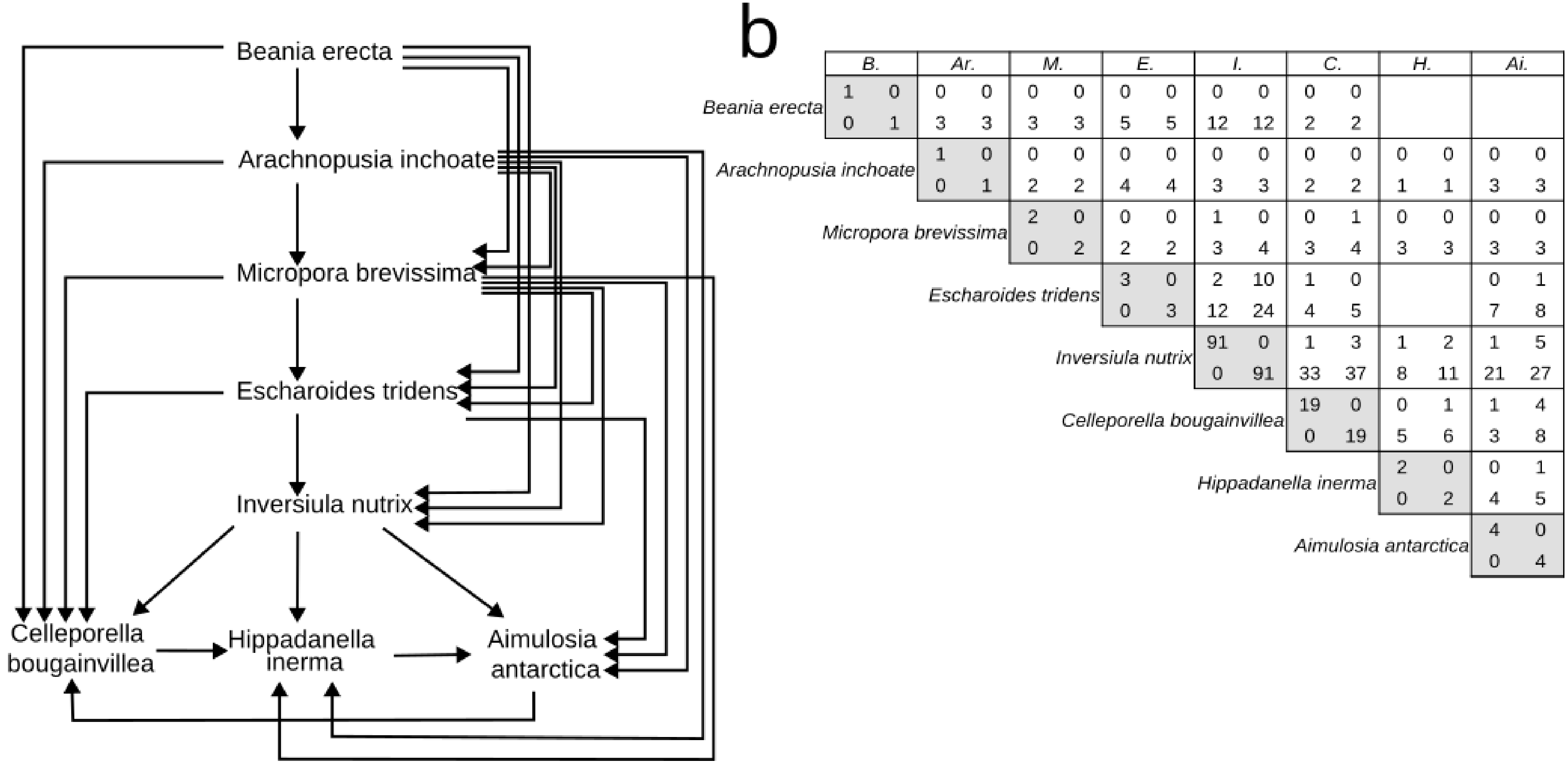

b

	B.		*Ar.*		*M.*		*E.*		*I.*		*C.*		*H.*		*Ai.*	
Beania erecta	1	0	0	0	0	0	0	0	0	0	0	0				
	0	1	3	3	3	3	5	5	12	12	2	2				
Arachnopusia inchoate			1	0	0	0	0	0	0	0	0	0	0	0	0	0
			0	1	2	2	4	4	3	3	2	2	1	1	3	3
Micropora brevissima					2	0	0	0	1	0	0	1	0	0	0	0
					0	2	2	2	3	4	3	4	3	3	3	3
Escharoides tridens							3	0	2	10	1	0			0	1
							0	3	12	24	4	5			7	8
Inversiula nutrix									91	0	1	3	1	2	1	5
									0	91	33	37	8	11	21	27
Celleporella bougainvillea											19	0	0	1	1	4
											0	19	5	6	3	8
Hippadanella inerma													2	0	0	1
													0	2	4	5
Aimulosia antarctica															4	0
															0	4

Supplementary Figure 1. Hierarchical ranking for example web from Signy Island, Antarctica

(a) Binarised version of the example web from Signy Island. The arrows point from the stronger competitor to the weaker competitor, based on who wins most of the observed contests for each pair. (b) shows the underlying data, the outcomes of all competitive interactions in the species-contact matrix. For each pair of species *i* (row) and *j* (column), the table contains one 2x2 square showing total number of contests (bottom right), wins by species *i* (bottom left), wins by species *j* (upper right) and draws (upper left). Grey squares indicate intraspecific contests.

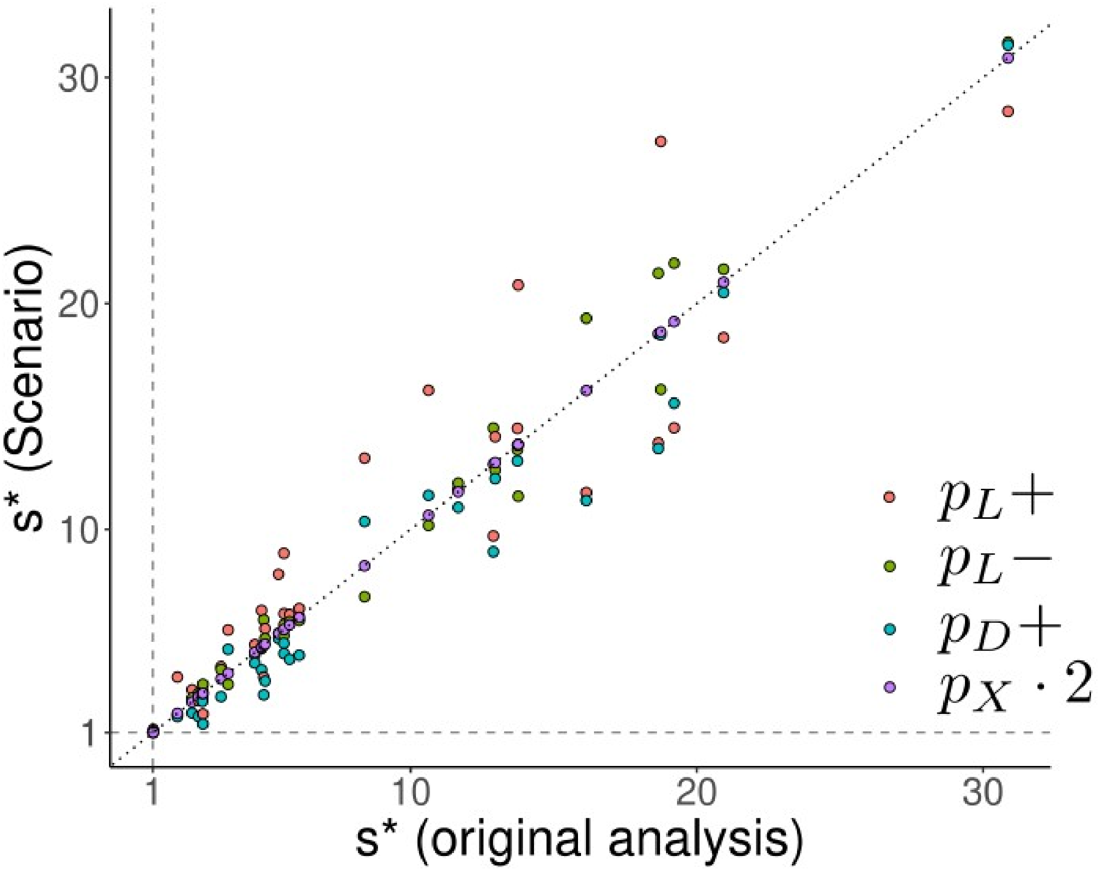

Supplementary Figure 2. Effect of varying cost values on stability of empirical networks

Dashed lines show the threshold between stable ($s* < 1$) and unstable systems ($s* > 1$). In the original analysis, cost values are defined as: p_L = -0.9, p_W = -0.1 and p_D = -0.2. In Scenario p_L-, the cost of a loss p_L is decreased to -0.4 and in Scenario p_L+, p_L is increased to -1.2. In Scenario , p_D+ the cost of a draw is increased to -0.3. Finally, in Scenario $p_x \cdot 2$ all cost values are doubled.

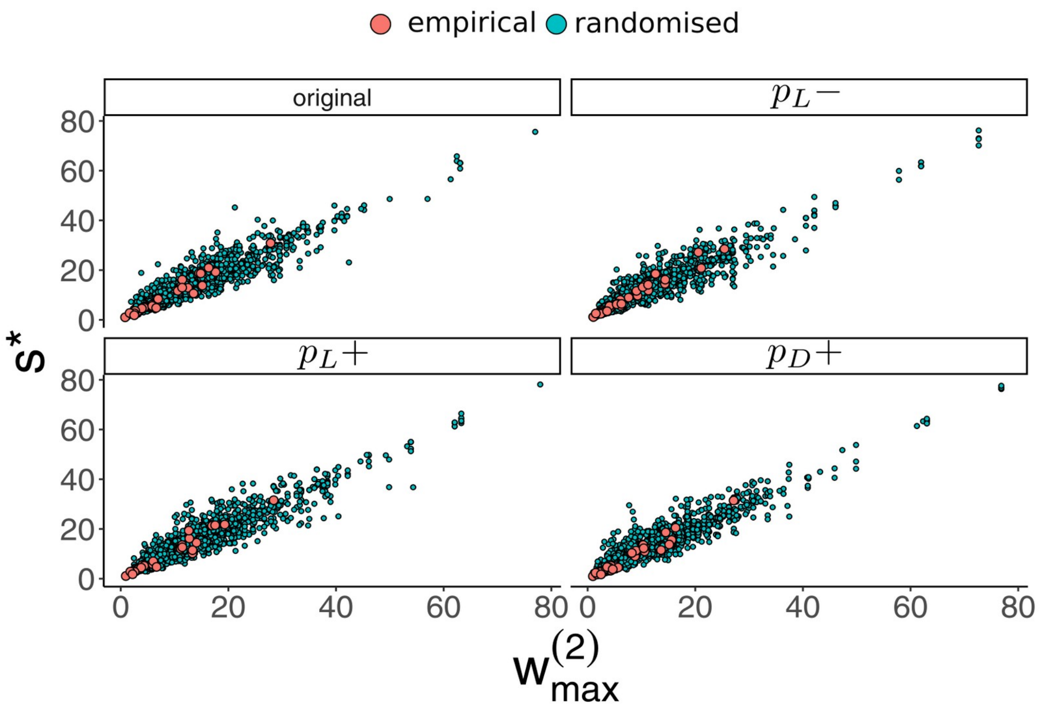

Supplementary Figure 3. The relation between stability (s*) and maximum weight of the 2-link loops ($w^{(2)}_{max}$) with varying cost values.

Varying cost values has a negligible effect on the relation between s^* and $w^{(2)}_{max}$ in the empirical systems, nor does it change the effect of randomisation. Scenarios are defined as in Supplementary Fig. 2. As Scenario $p_x \cdot 2$ did not have an effect on s^*, it is not included here.

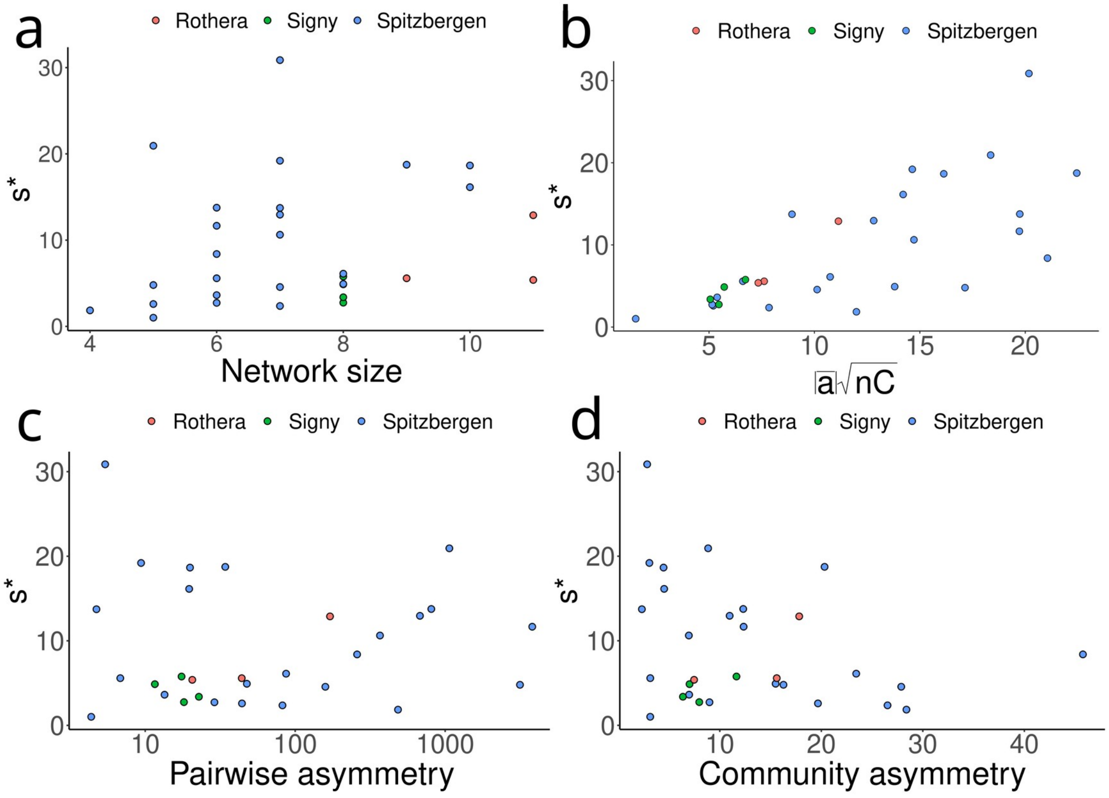

Supplementary Figure 4. Relationship between network size, complexity, asymmetry measures and stability (s*) in 30 empirical competition networks.

(a) Network size, describing the number of species (nodes) in the network vs. the critical amount of self-regulation s* ($y = 0.85x+3.06$, $R^2 = 0.044$, $p = 0.26$) . (b) May's complexity measure vs. critical self-regulation s^* ($y = 0.88x – 1.03$, $R^2 = 0.49$, $p < 0.001$). Complexity is defined here following (1) as $|\bar{a}|\sqrt{nC}$, with $\bar{a}$ being the mean link strength of the community matrix $\bar{A}$, the number of species n and connectance C. (c) Pairwise asymmetry ($y = 0.0004x + 9$, $R^2 = 0.003$, $p = 0.79$) and (d) Community asymmetry ($y = -0.15x + 10.86$, $R^2 = 0.061$, $p = 0.19$). Both asymmetry measures are calculated based on normalised interaction strengths.

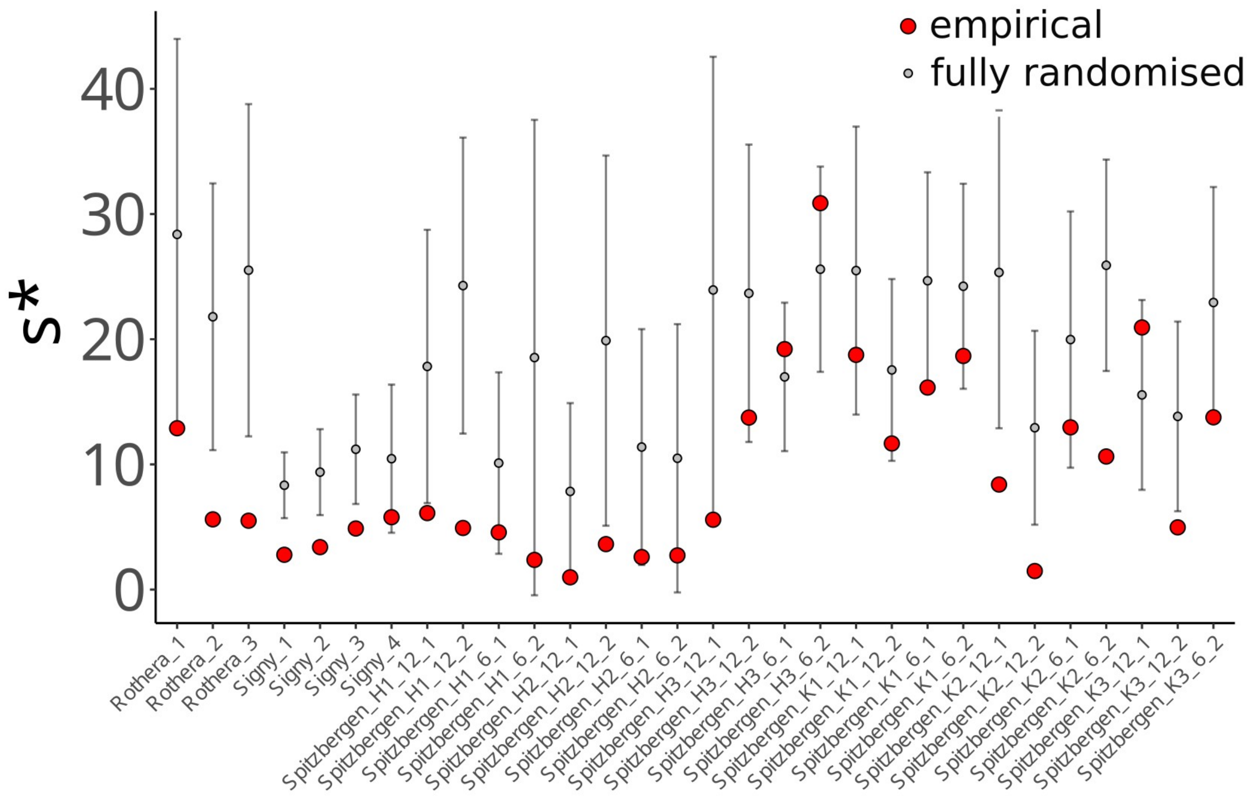

Supplementary Figure 5. Effect of full randomisation of the stability (s*) of raw empirical community matrices.

During full randomisation, all non-zero off-diagonal values are randomly reshuffled within the matrix, while the topology and complexity are preserved. Diagonals are kept in place. As the systems were not normalised, the randomisation thus affects the relationship between the off-diagonal and diagonal elements. The plot shows stability (s*) of the empirical systems (red points) compared to the mean s* (+/- standard deviation) of 1000 fully randomised versions of the same matrix (grey dots).

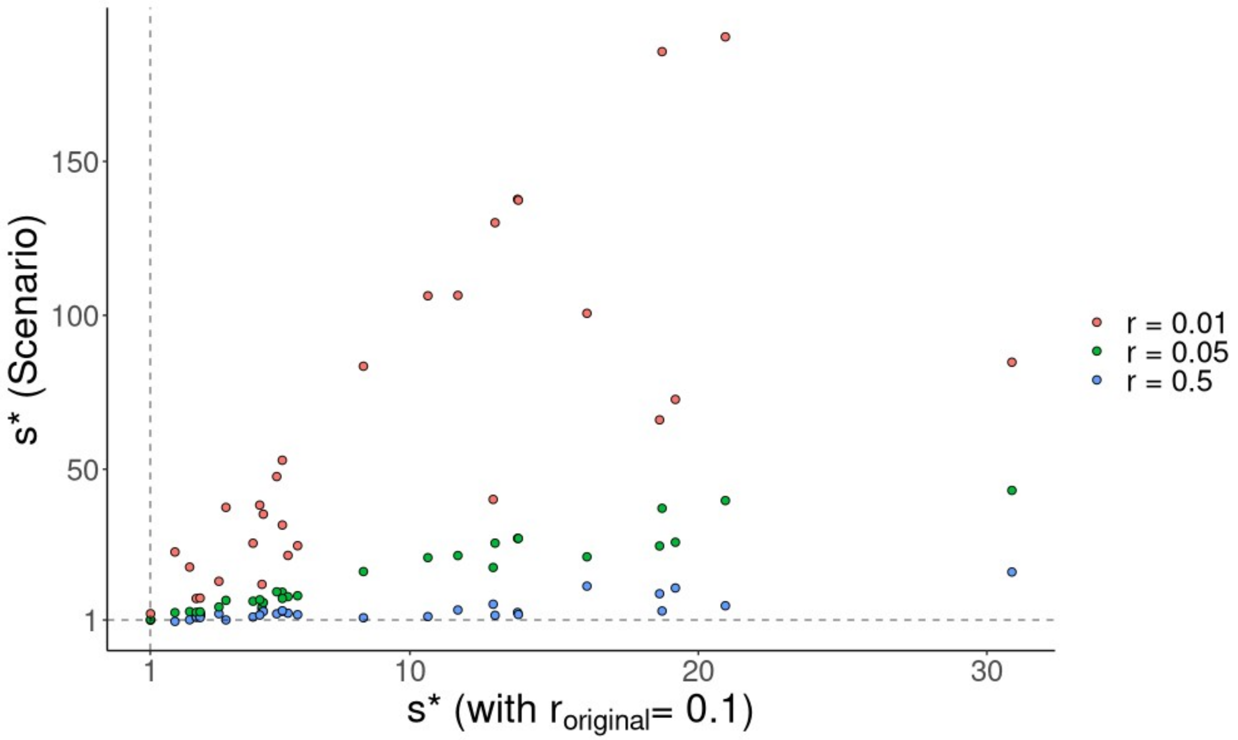

Supplementary Figure 6. Effect of replacing missing diagonal values in empirical networks on stability

Missing diagonal values were replaced by *r* multiplied by the average interaction strength. In the main analysis, *r* was set to 0.1. Dashed lines indicate $s^* = 1$, showing the boundary between stable ($s^* < 1$) and unstable systems ($s^* > 1$).

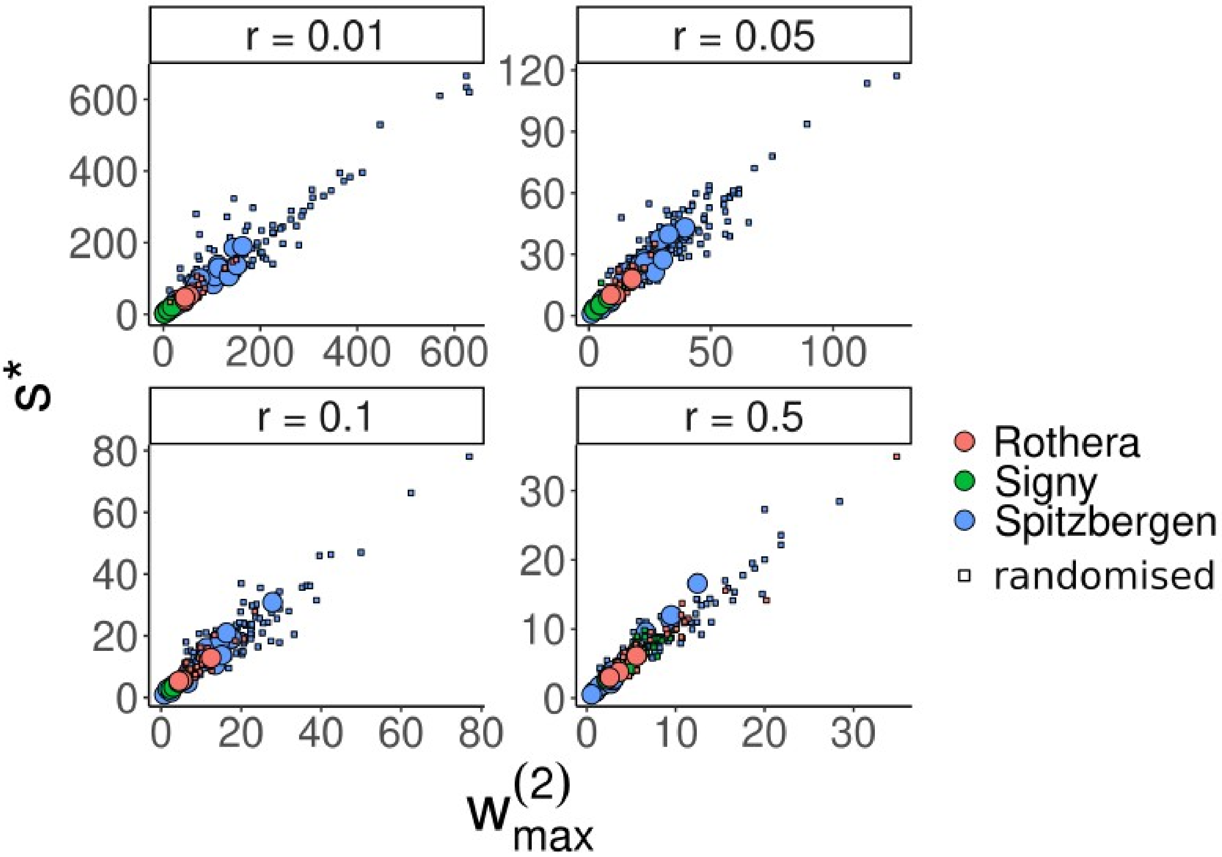

Supplementary Figure 7. Sensitivity of the relationship between $w^{(2)}_{max}$ and *s to replacement of missing diagonal values.**

Relation between maximum weight of 2-link loops ($w^{(2)}_{max}$) and critical self-regulation (*s**) for observed systems and their fully randomised counterparts under different scenarios of *r*. Scenarios for the replacement of missing diagonal values are as in Supplementary Fig. 6. In the main analysis *r* was set to 0.1. The choice of this value does not change the relationship between $w^{(2)}_{max}$ and *s**.

Supplementary Tables

					Energy loss matrix **F**		Jacobian matrix **A**			
Name	n	C	win-index	IC	pairwise asym-metry	Com. asym-metry	Pairwise asymmetry	Com. asymmetry	mean a_{ij}	$\mathrm{Re}(\lambda_d)$
Rothera 1*	11	0.45	0.63	-0.04	7.18	4.67	765.11	32.41	-0.33	0.37
Rothera 2*	9	0.47	0.64	-0.1	7.46	5.26	417.41	36.61	-0.12	0.07
Rothera 3*	11	0.55	0.69	0.01	7.37	5.29	173.6	18.95	-0.12	0.09
Signy 1*	8	0.89	0.86	0.01	6.54	3.35	28.65	8.98	-0.06	0.04
Signy 2	8	0.89	0.86	0.12	6.62	3.67	24.49	9.39	-0.05	0.03
Signy 3	8	0.89	0.87	0.1	6.81	4.12	22.87	7.62	-0.05	0.06
Signy 4	8	0.93	0.89	0.08	7.26	3.95	23.66	9.45	-0.05	0.06
Spitzb. H1-12-1	8	0.93	0.93	-0.12	8.16	6.26	43.38	16.43	-0.02	0.02
Spitzb. H1-12-2	8	0.96	0.96	0.12	8.01	6.39	28.59	15.21	-0.02	0.01
Spitzb. H1-6-1	7	0.95	0.94	0.04	7.34	5.97	28.41	12.38	-0.04	0.03
Spitzb. H1-6-2	7	0.95	0.96	-0.08	8.42	7.63	43.03	31.13	-0.05	0.02
Spitzb. H2-12-1	5	0.8	0.86	0.06	6.51	4.57	15.12	13.54	-0.05	0
Spitzb. H2-12-2	6	0.73	0.69	-0.02	4.97	3.49	25.66	14.57	-0.06	0.02
Spitzb. H2-6-1	5	0.6	0.7	0.03	4.76	2.54	11.27	8.01	-0.05	0.01
Spitzb. H2-6-2	6	0.73	0.76	0	6.03	4.26	12.4	8.07	-0.05	0.02
Spitzb. H3-12-1	6	0.93	0.83	0.03	6.07	2.59	14.61	8.29	-0.02	0.03
Spitzb. H3-12-2	7	0.86	0.73	-0.01	4.91	2.14	8.81	6.21	-0.02	0.03
Spitzb. H3-6-1	7	1	0.79	-0.11	4.89	2.31	33.13	2.62	-0.04	0.13
Spitzb. H3-6-2	7	0.95	0.85	-0.04	5.78	2.82	10.93	3.59	-0.01	0.03
Spitzb. K1-12-1	9	0.83	0.88	0.02	8.09	7.09	27.13	17.5	-0.03	0.06
Spitzb. K1-12-2	6	0.93	0.89	-0.02	6.39	3.17	20.05	5.55	-0.01	0.01

Spitzb. K1-6-1*	10	0.93	0.75	-0.03	4.29	2.72	9.82	4.29	-0.03	0.04
Spitzb. K1-6-2*	10	0.93	0.82	-0.07	5.5	2.73	11.9	4.29	-0.02	0.05
Spitzb. K2-12-1*	6	0.87	0.85	0.06	7.73	7.7	270.04	48.56	-0.03	0.02
Spitzb. K2-12-2*	4	0.83	0.86	-0.26	7.63	4.83	538.6	43.74	-0.03	0.01
Spitzb. K2-6-1	7	0.86	0.73	-0.09	5.54	3.22	227.16	12.02	-0.03	0.04
Spitzb. K2-6-2	7	0.86	0.76	-0.02	5.94	3.85	276.97	11.26	-0.04	0.04
Spitzb. K3-12-1	5	0.9	0.95	0.02	8.19	6.78	405.11	6.82	-0.16	0.42
Spitzb. K3-12-2	5	0.9	0.95	-0.28	9	9	810.75	11.89	-0.09	0.1
Spitzb. K3-6-2	6	0.93	0.95	-0.12	8.48	7.69	572.33	14.67	-0.09	0.13

Supplementary Table 1. Overview of network properties.

Networks marked with an asterisk have appeared in previous publications (see Methods), all unmarked datasets have not been published before. The table lists for all observed systems the following network properties: Size *n* (number of species), connectance *C* (number of actual links relative to the number of possible links), win-index (2), intransitivity index (3), pairwise and community asymmetry metrics for energy loss rates and interaction strengths. The win-index ranges from 0 to 1, with higher values indicating a stronger dominance of one competitor, an indication of a more hierarchical community structure. The intransitivity index (IC) measures the degree of intransitivity on a scale of -1 to +*n* with values from -1 to 1 indicating transitive structure, 1-2 weakly intransitive structure, 2-n intransitive structure. Pairwise asymmetry was calculated as the ratio of the stronger values vs. the weaker value of one interaction, averaged over all pairwise interactions in the network. Community asymmetry was calculated as the ratio of mean interaction strength in the lower diagonal and mean interaction strength in the upper diagonal. Mean interaction strength mean a_{ij} is the mean of all non-zero Jacobian elements. The

real part of the dominant eigenvalue $Re(\lambda_d)$ of the Jacobian matrix is a measure of stability: Positive values indicate that the Jacobian is unstable.

Name	s* (calculated numerically)	Re(λ_d) of normalised matrix $\bar{A}_0$)	Im($\left(\lambda_d\right)$) of the normalised matrix $\bar{A}_0$
Rothera 1	12.9	12.9	0
Rothera 2	5.7	5.6	1.15
Rothera 3	5.5	5.4	1.06
Signy 1	2.8	2.7	1.09
Signy 2	3.4	3.4	0
Signy 3	4.9	4.9	0
Signy 4	5.8	5.8	0
Spitzbergen H1-12-1	6.2	6.1	0
Spitzbergen H1-12-2	5	4.9	0
Spitzbergen H1-6-1	4.6	4.6	0
Spitzbergen H1-6-2	2.4	2.4	0
Spitzbergen H2-12-1	1	1	0.33
Spitzbergen H2-12-2	3.7	3.6	0
Spitzbergen H2-6-1	2.6	2.6	0
Spitzbergen H2-6-2	2.8	2.7	0
Spitzbergen H3-12-1	5.6	5.6	0
Spitzbergen H3-12-2	13.8	13.7	0
Spitzbergen H3-6-1	19.3	19.2	0
Spitzbergen H3-6-2	30.9	30.9	0
Spitzbergen K1-12-1	18.8	18.7	0
Spitzbergen K1-12-2	11.7	11.7	0
Spitzbergen K1-6-1	16.2	16.1	0
Spitzbergen K1-6-2	18.7	18.7	0
Spitzbergen K2-12-1	8.4	8.4	0
Spitzbergen K2-12-2	1.5	1.9	0.97
Spitzbergen K2-6-1	13	13	0
Spitzbergen K2-6-2	10.7	10.6	3.08
Spitzbergen K3-12-1	21	20.9	0
Spitzbergen K3-12-2	5	4.8	1.59
Spitzbergen K3-6-2	13.8	13.8	0

Supplementary Table 2. Comparison between critical self-regulation s* and the maximum eigenvalues of normalised community matrices.

The critical self-regulation s* is the factor by which the observed intraspecific interaction strengths need to be multiplied in order to reach stability. S*< 1 indicates stability while s*> 1 indicates instability. *s** of a matrix was calculated by multiplying the matrix diagonal with a

(positive) control parameter $s = 1$, and then continually increasing s until all the eigenvalues of the matrix have negative real parts. Re(λ_d) is the maximum real part of the eigenvalues of the normalised matrix $\bar{A}$ with diagonals set at zero, $\bar{A}_0$. Normalised matrices are obtained by dividing all off-diagonal elements a_{ij} by the absolute value of their respective diagonal element a_{ii}. The stability properties of the normalised matrices were found to be equal or almost equal to that of the raw empirical Jacobian matrix (see Methods, (4,5)). For the normalised matrix $\bar{A}$, with diagonal elements -1, s* is, by definition, equivalent to the maximum real part of the eigenvalues of $\bar{A}_0$. Im(λ_d) is the imaginary part of the eigenvalue with the maximum real part λ_d.

Name	Pairwise asymmetry	Community asymmetry	Mean(a_{ij})	Re(λ_d) of $\bar{A}_0$
Rothera 1	170.8	17.8	-4.98	12.9
Rothera 2	44	15.6	-3.69	5.6
Rothera 3	20.6	7.5	-2.99	5.4
Signy 1	18.1	8	-2.05	2.7
Signy 2	22.8	6.4	-1.89	3.4
Signy 3	11.6	7	-2.14	4.9
Signy 4	17.5	11.6	-2.47	5.8
Spitzbergen H1-12-1	87	23.4	-3.94	6.1
Spitzbergen H1-12-2	47.6	15.5	-4.97	4.9
Spitzbergen H1-6-1	159	27.9	-3.92	4.6
Spitzbergen H1-6-2	82.3	26.5	-3.04	2.4
Spitzbergen H2-12-1	4.4	3.1	-0.76	1
Spitzbergen H2-2-2	13.5	7	-2.57	3.6
Spitzbergen H2-6-1	44.2	19.7	-3.01	2.6
Spitzbergen H2-6-2	28.9	9	-2.45	2.7
Spitzbergen H3-12-1	6.8	3.1	-2.79	5.6
Spitzbergen H3-12-2	4.7	2.3	-3.65	13.7
Spitzbergen H3-6-1	9.4	3.1	-5.53	19.2
Spitzbergen H3-6-2	5.4	2.8	-7.81	30.9
Spitzbergen K1-12-1	34.2	20.3	-8.2	18.7
Spitzbergen K1-12-2	3809.8	12.3	-8.33	11.7
Spitzbergen K1-6-1	19.6	4.5	-4.65	16.1
Spitzbergen K1-6-2	19.9	4.5	-5.28	18.7
Spitzbergen K2-12-1	258.8	45.8	-9.23	8.4
Spitzbergen K2-12-2	485	28.4	-6.57	1.9
Spitzbergen K2-6-1	679.2	11	-5.23	13
Spitzbergen K2-6-2	367.8	6.9	-6.01	10.6
Spitzbergen K3-12-1	1066.8	8.8	-8.65	20.9
Spitzbergen K3-12-2	3155.2	16.3	-8.08	4.8
Spitzbergen K3-6-2	808.5	12.3	-8.34	13.8

Supplementary Table 3. Properties of normalised community matrices

The community matrices were normalised by dividing each row in the matrix by the absolute value of its corresponding diagonal term, following (4). This translates the diagonal structure into the off-diagonal structure and enables the transparent comparison of patterns of interaction strengths in community matrices with varying diagonal elements. The table shows community and pairwise asymmetry measures, the mean interaction strength as well as the dominant

eigenvalue of the matrix with diagonals set to 0, which corresponds to s* of the raw matrix (see Table S3).

A: Effect of network manipulations on pairwise asymmetry						
Name	Empirical	Randomisations			Maximised	
		Minimal	Weak	Full	Pairwise asymmetry	Community asymmetry
Rothera 1	170.8	170.8	170.8	143.1	1105.3	1105.3
Rothera 2	44	44	44	65.7	235.9	235.9
Rothera 3	20.6	20.6	20.6	19.4	57.7	57.7
Signy 1	18.1	18.1	18.1	13	36.3	36.3
Signy 2	22.8	22.8	22.8	13.5	39.3	39.3
Signy 3	11.6	11.6	11.6	9.9	28.1	28.1
Signy 4	17.5	17.5	17.5	15.7	59	59
Spitzbergen H1-12-1	87	87	87	55.8	265.8	265.8
Spitzbergen H1-12-2	47.6	47.6	47.6	23.6	88.8	88.8
Spitzbergen H1-6-1	159	159	159	135	866.3	866.3
Spitzbergen H1-6-2	82.3	82.3	82.3	49.1	257.8	257.8
Spitzbergen H2-12-1	4.4	4.4	4.4	3.6	5.5	5.5
Spitzbergen H2-12-2	13.5	13.5	13.5	6.6	15.6	15.6
Spitzbergen H2-6-1	44.2	44.2	44.2	27.9	77.8	77.8
Spitzbergen H2-6-2	28.9	28.9	28.9	13	34.2	34.2
Spitzbergen H3-12-1	6.8	6.8	6.8	11.3	29.1	29.1
Spitzbergen H3-12-2	4.7	4.7	4.7	20.3	73.4	73.4
Spitzbergen H3-6-1	9.4	9.4	9.4	39	145.5	145.5
Spitzbergen H3-6-2	5.4	5.4	5.4	24.4	112.7	112.7
Spitzbergen K1-12-1	34.2	34.2	34.2	33.7	165.3	165.3
Spitzbergen K1-12-2	3809.8	3809.8	3809.8	2014	10789.1	10789.1
Spitzbergen K1-6-1	19.6	19.6	19.6	23.1	86.3	86.3
Spitzbergen K1-6-2	19.9	19.9	19.9	25.1	101.9	101.9

Spitzbergen K2-12-1	258.8	258.8	258.8	208.5	1176.2	1176.2
Spitzbergen K2-12-2	485	485	485	155.7	485.5	485.5
Spitzbergen K2-6-1	679.2	679.2	679.2	546	4586	4586
Spitzbergen K2-6-2	367.8	367.8	367.8	2000.2	9075.6	9075.6
Spitzbergen K3-12-1	1066.8	1066.8	1066.8	1385.1	6541.8	6541.8
Spitzbergen K3-12-2	3155.2	3155.2	3155.2	1129.9	4503.4	4503.4
Spitzbergen K3-6-2	808.5	808.5	808.5	299.7	1345.6	1345.6

B: Effect of network manipulations on $w_{max}^{(2)}$						
Name	Empirical	Randomisations			Maximised	
		Minimal	Weak	Full	Pairwise asymmetry	Community asymmetry
Rothera 1	12.5	12.5	12.5	16.4	1.2	1.2
Rothera 2	5.4	5.4	5.4	10.3	1.1	1.1
Rothera 3	4.5	4.5	4.5	8.1	1.8	1.8
Signy 1	2.3	2.3	2.3	5.6	1.2	1.2
Signy 2	3.1	3.1	3.1	5	1.3	1.3
Signy 3	4.5	4.5	4.5	6.1	1.4	1.4
Signy 4	5.7	5.7	5.7	7.9	1.1	1.1
Spitzbergen H1-12-1	5.8	5.8	5.8	13.3	1.1	1.1
Spitzbergen H1-12-2	4.3	4.3	4.3	16.1	2	2
Spitzbergen H1-6-1	3.9	3.9	3.9	11.5	1	1
Spitzbergen H1-6-2	2.2	2.2	2.2	8.9	0.8	0.8
Spitzbergen H2-12-1	0.8	0.8	0.8	1.2	0.8	0.8
Spitzbergen H2-12-2	2.7	2.7	2.7	5.7	1.8	1.8
Spitzbergen H2-6-1	2.5	2.5	2.5	5.3	1.7	1.7
Spitzbergen H2-6-2	1.7	1.7	1.7	5.5	1.3	1.3
Spitzbergen H3-12-1	5.9	5.9	5.9	6.2	1.9	1.9
Spitzbergen H3-12-2	11.3	11.3	11.3	8.8	2.5	2.5
Spitzbergen H3-6-1	17.6	17.6	17.6	14	4.1	4.1
Spitzbergen H3-6-2	27.8	27.8	27.8	21.4	3.8	3.8
Spitzbergen K1-12-1	14.5	14.5	14.5	29.3	2.8	2.8
Spitzbergen K1-12-2	10.6	10.6	10.6	21.4	3.6	3.6
Spitzbergen K1-6-1	11.4	11.4	11.4	15.1	2.4	2.4
Spitzbergen K1-6-2	14.9	14.9	14.9	17.4	2.7	2.7

B: Effect of network manipulations on $w_{max}^{(2)}$						
Name	Empirical	Randomisations			Maximised	
		Minimal	Weak	Full	Pairwise asymmetry	Community asymmetry
Spitzbergen K2-12-1	6.9	6.9	6.9	23.2	2	2
Spitzbergen K2-12-2	2.5	2.5	2.5	9.5	1.8	1.8
Spitzbergen K2-6-1	11.4	11.4	11.4	15.3	1	1
Spitzbergen K2-6-2	13.5	13.5	13.5	16.4	2.2	2.2
Spitzbergen K3-12-1	16.3	16.3	16.3	17.7	3.3	3.3
Spitzbergen K3-12-2	6.6	6.6	6.6	17.9	2.5	2.5
Spitzbergen K3-6-2	15.2	15.2	15.2	21.7	2.7	2.7

C: Effect of network manipulations on s*						
Name	Empirical	Randomisations			Maximised	
		Minimal	Weak	Full	Pairwise asymmetry	Community asymmetry
Rothera 1	12.9	12.2	12.7	18.4	8.8	5.4
Rothera 2	5.6	6.8	7.3	11.3	5.4	3.5
Rothera 3	5.4	7	7	10	5.3	4.5
Signy 1	2.7	3.3	3.9	6.3	3.3	2.3
Signy 2	3.4	3.7	3.8	5.7	3	2.4
Signy 3	4.9	4.9	5	6.7	3.6	2.6
Signy 4	5.8	6	6.4	8.8	4.9	3.1
Spitzbergen H1-12-1	6.1	5.9	8.8	14.7	8.1	4
Spitzbergen H1-12-2	4.9	6.6	10.3	17.4	9.8	5.3
Spitzbergen H1-6-1	4.6	5.4	7.3	12.9	6.8	3.1
Spitzbergen H1-6-2	2.4	3.8	5.3	9.8	5	2.6
Spitzbergen H2-12-1	1	1.1	1.1	1.4	1	0.9
Spitzbergen H2-12-2	3.6	3.6	3.7	6.1	3.4	2.6
Spitzbergen H2-6-1	2.6	3	2.9	5.6	2.4	1.9
Spitzbergen H2-6-2	2.7	3.1	3	5.7	2.8	2.2
Spitzbergen H3-12-1	5.6	6.5	6.5	6.6	3.5	2.9
Spitzbergen H3-12-2	13.7	12.6	12.5	9.7	5	4.1
Spitzbergen H3-6-1	19.2	19.2	18.3	14.9	7.5	5.5
Spitzbergen H3-6-2	30.9	28.2	28.4	22.9	12.5	8.1
Spitzbergen K1-12-1	18.7	15.9	20.7	32.7	17.8	8.2
Spitzbergen K1-12-2	11.7	13.5	13.8	22.2	11.2	7.1
Spitzbergen K1-6-1	16.1	15.2	15	17.3	9	6
Spitzbergen K1-6-2	18.7	17.9	18	19.9	10.9	7.1

C: Effect of network manipulations on s*						
Name	Empirical	Randomisations			Maximised	
		Minimal	Weak	Full	Pairwise asymmetry	Community asymmetry
Spitzbergen K2-12-1	8.4	7.8	13.5	25	12	4.8
Spitzbergen K2-12-2	1.9	2.7	4	9.8	3.9	2.6
Spitzbergen K2-6-1	13	11.4	12.2	16.6	8.4	4.1
Spitzbergen K2-6-2	10.6	14.5	14.3	17.9	9.8	5.9
Spitzbergen K3-12-1	20.9	17.8	17.1	18.4	8.4	6.2
Spitzbergen K3-12-2	4.8	7.1	9.6	18.5	9	4.6
Spitzbergen K3-6-2	13.8	13.1	15	23	11.7	6.2

Supplementary Table 4. Effect of manipulations on pairwise asymmetry, the weight of the strongest 2-link loops $w^{(2)}_{max}$ and stability (s*) for all webs.

Our manipulations destroyed the internal feedback structure while preserving the average interaction strength, topology and complexity of the network (in the sense of (1)). For manipulated networks, the values shown are the average of 1000 manipulated matrices. *Weak randomisation* preserves pairs of interaction strengths but randomises their location, preserving pairwise asymmetry but destroying community asymmetry. *Full randomisation* randomly reshuffles link strengths within the matrices, destroying both community and pairwise asymmetry. In the other two manipulations, links were reordered to restore asymmetries: In "*pairwise asymmetry*", links are order form pairs that are as asymmetric as possible. In "community+ pairwise asymmetry", community asymmetry was also maximised by placing all strong links on one side of the diagonal. The effects of randomisation on *s** varies, due to different levels of pairwise and community asymmetry in the empirical matrices. For all calculations, the normalised matrices $\bar{A}$ were used.

Supplementary References

1. May R. Will a large complex system be stable? Nature. 1972;238:413–4.

2. Tanaka M, Nandakumar K. Measurement of the degree of intransitivity in a community of sessile organisms. J Exp Mar Biol Ecol. 1994 Sep;182(1):85–95.

3. Gallien L, Landi P, Hui C, Richardson D. Emergence of weak-intransitive competition through adaptive diversification and eco-evolutionary feedbacks. Allan E, editor. J Ecol. 2018 May;106(3):877–89.

4. Neutel AM, Thorne M. Interaction strengths in balanced carbon cycles and the absence of a relation between ecosystem complexity and stability. Adler F, editor. Ecol Lett. 2014 Jun;17(6):651–61.

5. Thorne MAS, Forgoston E, Billings L, Neutel AM. Matrix Scaling and Tipping Points. SIAM J Appl Dyn Syst. 2021 Jan 1;20(2):1090–103.
